# Targeting oncogenic MAGEA6 sensitizes triple negative breast cancer to doxorubicin through its autophagy and ferroptosis by stabling AMPKα1

**DOI:** 10.1038/s41420-024-02196-9

**Published:** 2024-10-06

**Authors:** Hui Zhu, Cheng-wei Jiang, Wen-long Zhang, Zhao-ying Yang, Guang Sun

**Affiliations:** 1 Department of Breast Surgery, China–Japan Union Hospital of Jilin University, 130033 Changchun, Jilin China; 2 Department of Pathology, China–Japan Union Hospital of Jilin University, 130033 Changchun, Jilin China; 3 Department of Hematology and Oncology, China–Japan Union Hospital of Jilin University, 130033 Changchun, Jilin China

**Keywords:** Breast cancer, Molecular biology

## Abstract

Melanoma-associated antigen A6 (MAGEA6) is well known to have oncogenic activity, but the underlying mechanisms by which it regulates tumor progression and chemo-resistance, especially in triple-negative breast cancer (TNBC), have been unknown. In the study, the differential expression genes (DEGs) in TNBC tumor tissues and TNBC-resistant tumor tissues were analyzed based on TCGA and GEO datasets. MAGEA6, as the most significantly expressed gene, was analyzed by RT-qPCR, western blotting and immunohistochemistry assay in TNBC cell lines and tumor tissues. The potential mechanisms that influence chemo-resistance were also evaluated. Results displayed that MAGEA6 was highly expressed in TNBC and involved in drug resistance. MAGEA6 silencing enhanced the chemo-sensitivity of TNBC to doxorubicin (DOX) in vitro and in vivo, as determined by decreasing IC_50_ value, proliferation and invasion capacity, and triggering apoptosis. Mechanistically, it was shown that MAGEA6 depletion sensitized TNBC to DOX via regulating autophagy. Ubiquitination assay displayed that knockdown of MAGEA6 decreased the AMPKα1 ubiquitination, thereby elevating the levels of AMPKα1 and p-AMPKα in TNBC cells. Importantly, AMPK inhibitor (Compound C) can reduce the LC3II/I level induced by sh-MAGEA6, indicating that sh-MAGEA6 activated AMPK signaling through suppressing AMPKα1 ubiquitination and then facilitated autophagy in TNBC. Furthermore, we also observed that AMPK is required for SLC7A11 to regulate ferroptosis, and supported the crux roles of MAGEA6/AMPK/SLC7A11-mediated ferroptosis on modulating DOX sensitivity in TNBC cells. These findings indicated that targeting MAGEA6 can enhance the chemo-sensitivity in TNBC via activation of autophagy and ferroptosis; its mechanism involves AMPKα1-dependent autophagy and AMPKα1/SLC7A11-induced ferroptosis.

## Introduction

Triple-negative breast cancer (TNBC), as the most invasive subtype, accounts for ~10–20% of breast cancer [1]. Regarding characteristics, TNBC patients present the negative expression of progesterone receptor (PR), estrogen receptor alpha (ERα), and epidermal growth factor receptor 2 (HER2), consequently, reducing the sensitivity to therapies and increasing the treatment difficulties [2]. In clinical practice, chemotherapies have been extensively applied for TNBC patients [3]. However, chemo-resistance mainly occurs in almost 50% of patients, which limits clinical efficacy and results in bad prognosis [4]. As a consequence, an in-depth understanding of how to solve the problem of chemo-resistance is urgently needed in TNBC.

As we know, dysfunction of cell processes, such as enrichment of cancer stem cells (CSCs) [5], autophagy [6], and ferroptosis [7], are associated with chemo-resistance. In TNBC, several evidences have demonstrated that targeting autophagy could increase the sensitivity of TNBC cells to chemotherapeutic agents [8, 9]. Generally, autophagy is closely related to ferroptosis. Ferroptosis, an iron-dependent type of cell death, has been shown to be involved in resistance to TNBC [10, 11]. Thus, autophagy/ferroptosis may be a therapeutic target for overcoming TNBC resistance.

Several genes have been reported to modulate autophagy, including the members of the melanoma-associated antigens MAGE-family [12]. As an oncogene, MAGEA is consistent with a role in tumor progression, and high MAGEA expression has been associated with poor prognosis. Due to its high tumor specificity, targeting MAGEA genes has been appreciated as a promising immunotherapy agent [13]. Interactions between MAGEA and resistance. There is evidence showing that MAGEA is up-regulated in resistant tumor cells, including breast cancer [14–16]. MAGEA6, as a member of the MAGEA family, has been verified to be a hub gene MAGEA6 involved in chemo-resistance and survival prognosis of breast cancer [17]. However, in TNBC, the functional effects of MAGEA6 on chemo-resistance and potential mechanisms have not yet been fully revealed.

Here, we present evidence for a regulatory axis engaged in TNBC chemo-resistance that MAGEA6 mediated the degradation of AMPKα1 through ubiquitination, influences the autophagy and ferroptosis, and then enhances chemo-resistance.

## Results

### MAGEA6 was highly expressed and involved in drug resistance in TNBC

**Fig. 1:**
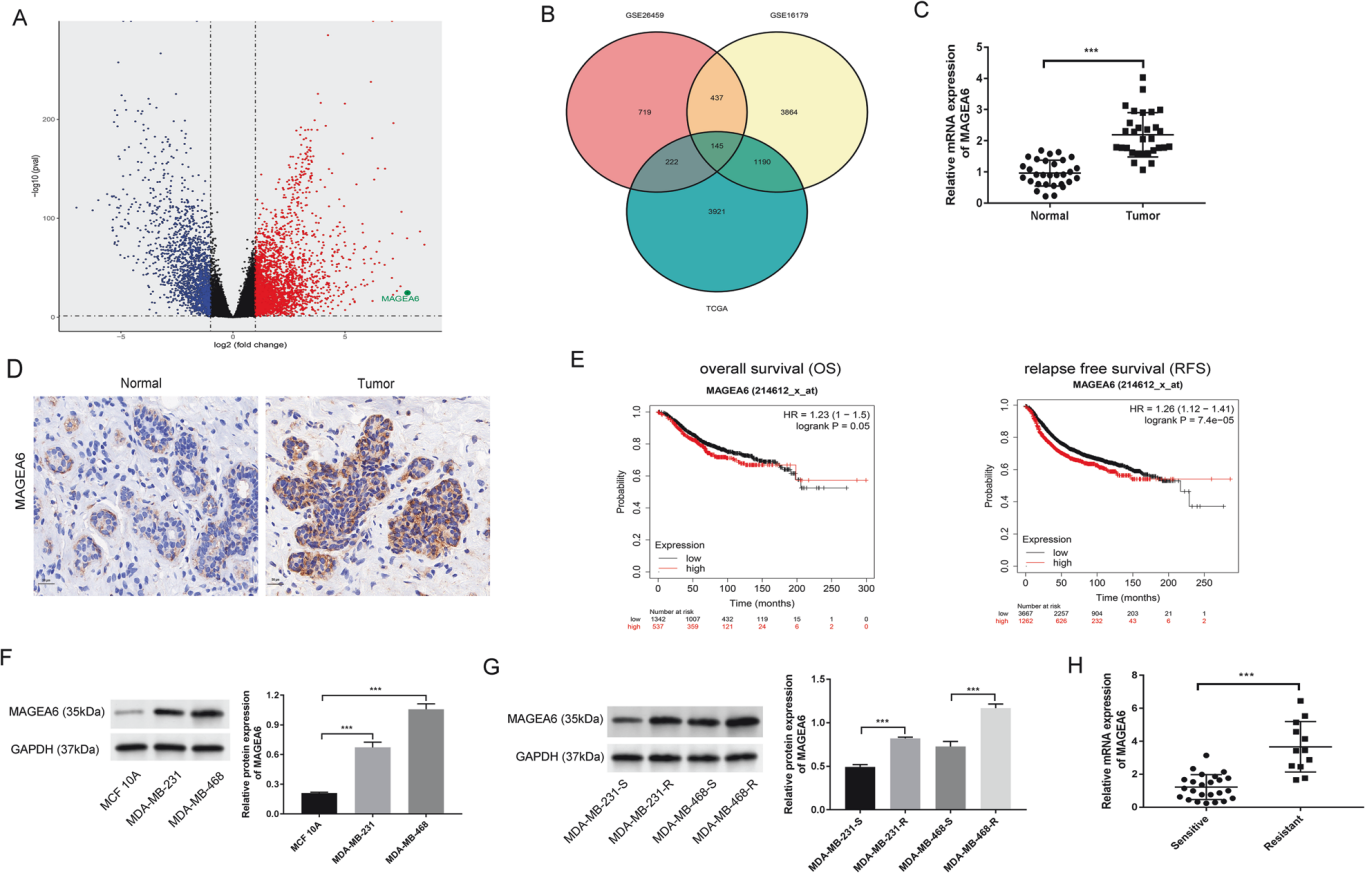
MAGEA6 was highly expressed and involved in drug resistance in TNBC. **A** Volcano plot showed the DEGs of breast cancer based on TCGA datasets. **B** The pivotal genes influencing the cancer progression (TCGA) and drug resistance (GSE26459/GSE16179) were shown by the Venn diagram. **C**, **D** The MAGEA6 expression in clinical samples was tested by RT-qPCR and immunohistochemistry assay. **E** The prognosis induced by MAGEA6 expression was analyzed by Kaplan–Meier in patients with breast cancer; The MAGEA6 expression in TNBC cell lines (MDA-MB-231 and MDA-MB-468) (**F**) and DOX-resistant TNBC cells (MDA-MB-231-R and MDA-MB-468-R) (**G**) were tested by western blotting. **H** RT-qPCR assay was employed to test the MAGEA6 expression in DOX-resistant and DOX-sensitive TNBC tissues; ^***^P < 0.001; Scale bar = 50 μm. Data in vitro were obtained from three independent experiments. DEGs differential expression genes, DOX doxorubicin, TNBC triple-negative breast cancer.

To identify the DEGs in breast cancer based on TCGA datasets, we found the top dysregulated genes, as shown in the volcano plot (Fig. 1A). Additionally, the pivotal gene influencing the effect of drug resistance, an analysis of two GEO datasets was undertaken. As presented in Fig. 1B, a total of 145 DEGs were found to be involved in both cancer progression and drug resistance; among them, MAGEA6 log_2_ FC was most significantly expressed in TCGA, and significant differences were also found in GSE26459/GSE16179 (suggesting involvement in drug resistance). In clinical samples and TNBC cell lines, the high expression of MAGEA6 was further verified (Fig. 1C, D, F). Kaplan–Meier plots showed that patients with high expression of MAGEA6 had shorter OS and RFS (Fig. 1E). After constructing a resistant TNBC cell line, we found that MAGEA6 was up-regulated in doxorubicin (DOX)-resistant TNBC cells (MDA-MB-231-R, MDA-MB-468-R) (Figs. S1 and 1G). In line, MAGEA6 was notably higher in DOX-resistant TNBC tissues than in sensitive TNBC tissues (Fig. 1H).

### MAGEA6 silencing enhanced the chemo-sensitivity to DOX in TNBC cells

**Fig. 2:**
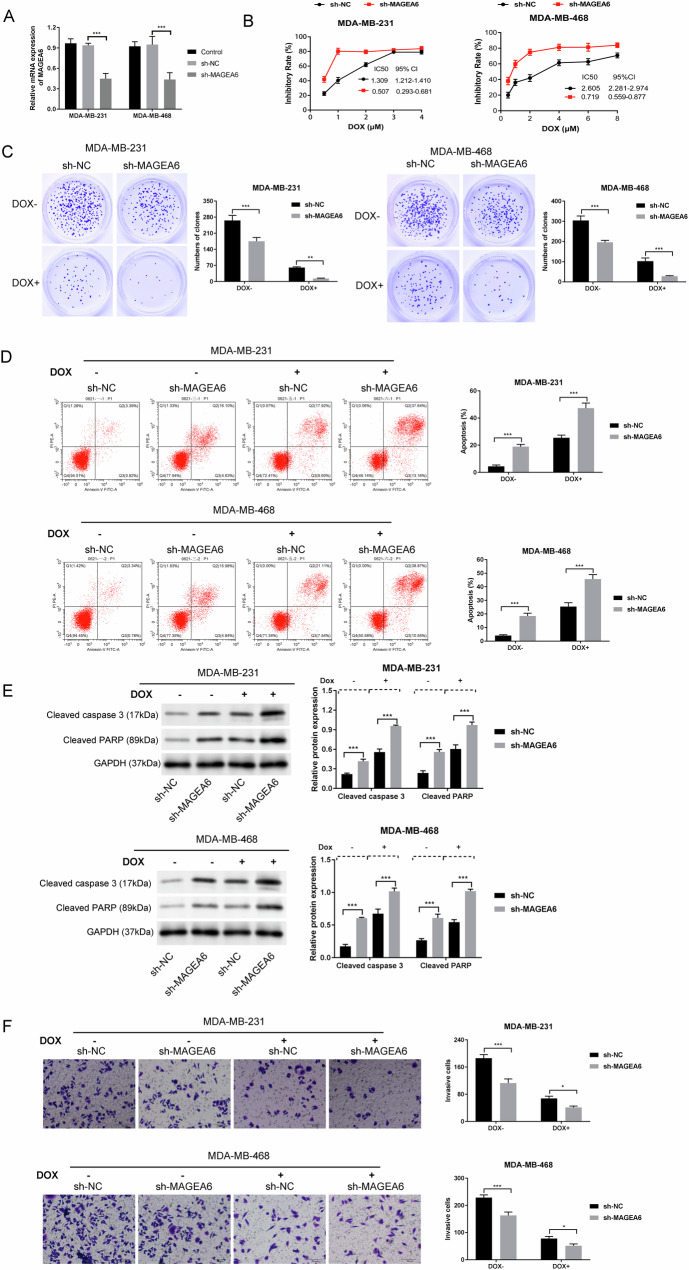
MAGEA6 silencing enhanced the chemo-sensitivity to DOX in TNBC cells. **A** Transfection efficacy was verified by RT-qPCR assay. **B** The influences of sh-MAGEA6 on IC_50_ value in MDA-MB-231 and MDA-MB-468 cells were determined by MTT assay. **C** Colony formation was utilized to test the proliferation ability; Flow cytometry (**D**) and Western blotting (**E**) showed the roles of sh-MAGEA6 on apoptosis in both MDA-MB-231 and MDA-MB-468 cells treated with/without DOX. **F** Transwell assay was utilized to test the roles of sh-MAGEA6 on the invasion ability of MDA-MB-231 and MDA-MB-468 cells treated with/without DOX; *P < 0.05, **P < 0.01, ***P < 0.001; Data were obtained from three independent experiments. DOX doxorubicin, TNBC triple-negative breast cancer.

To further verify the function of MAGEA6 on chemo-sensitivity in TNBC cells, MDA-MB-231 and MDA-MB-468 were transfected with sh-MAGEA6 vector or sh-NC. Results showed that shRNA successfully knocked out the MAGEA6 in MDA-MB-231 and MDA-MB-468 cells (Fig. 2A). MTT assay demonstrated that reduced MAGEA6 obviously improved the chemo-sensitivity of TNBC cells to DOX, with a remarkable decrease in the IC_50_ value (Fig. 2B). Colony formation assay was line with the MTT findings (Fig. 2C). Apart from, we found that MAGEA6 silencing notably facilitated DOX-induced apoptosis (Fig. 2D, E). Subsequent transwell assay also demonstrated that MAGEA6 knockdown obviously elevated the killing effect of DOX in both MDA-MB-231 and MDA-MB-468 cells, as evidenced by reducing the invasion ability (*P* < 0.001) (Fig. 2F). Aforementioned findings indicated that silencing of MAGEA6 enhanced the DOX sensitivity of TNBC.

### MAGEA6 regulated tumor chemo-sensitivity via negatively regulating autophagy

**Fig. 3:**
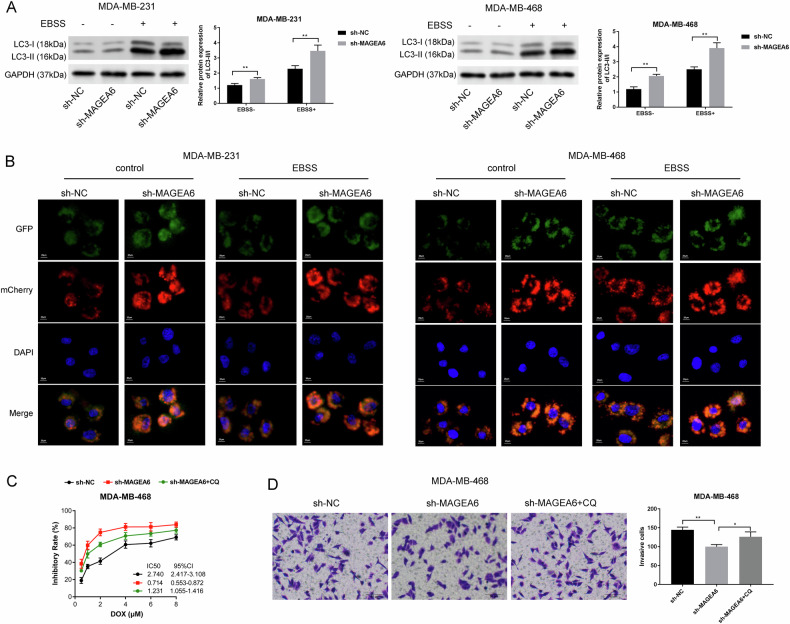
MAGEA6 regulated tumor chemo-sensitivity via negatively regulating autophagy. **A** Western blotting displayed that sh-MAGEA6 remarkably increased the amount of LC3-II/LC3I in MDA-MB-231 and MDA-MB-468 cells under conditions of EBSS (autophagy activator). **B** mRFP-GFP LC3 autophagy assay was employed to detect the influences of sh-MAGEA6 on autophagosomes (mCherry+GFP+, yellow); MTT assay (**C**) and Transwell assay (**D**) presented that CQ (autophagy inhibitor) can reverse the decreases of IC_50_ value and invasion ability caused by MAGEA6 depletion; ^*^P < 0.05, **P < 0.01; Data were obtained from three independent experiments; Scale bar = 20 μm. EBSS Earle’s balanced salt solution, CQ chloroquine.

At first, Gene Ontology (GO)—the biological process for MAGEA6 shows that it is involved in the negative regulation of autophagy (GO: 0010507). Next, we tested its effects in TNBC cells that activate autophagy under EBSS conditions. Results displayed that MAGEA6 silencing remarkably increased the amount of LC3-II/LC3I in MDA-MB-231 and MDA-MB-468 cells treated with EBSS (Fig. 3A). In line, knockdown of MAGEA6 also increased autophagosomes marked with yellow (mCherry+GFP+) (Fig. 3B). To further verify whether drug resistance is affected by autophagy, autophagy inhibitor (chloroquine, CQ) was added. Results uncovered that CQ can reverse the decreases of IC_50_ value and invasion ability caused by MAGEA6 depletion (Fig. 3C, D). Above all, we believed that MAGEA6 regulates tumor chemo-sensitivity via negatively modulating autophagy.

### Inhibition of AMPK by MAGEA6 impacted the autophagy of TNBC

**Fig. 4:**
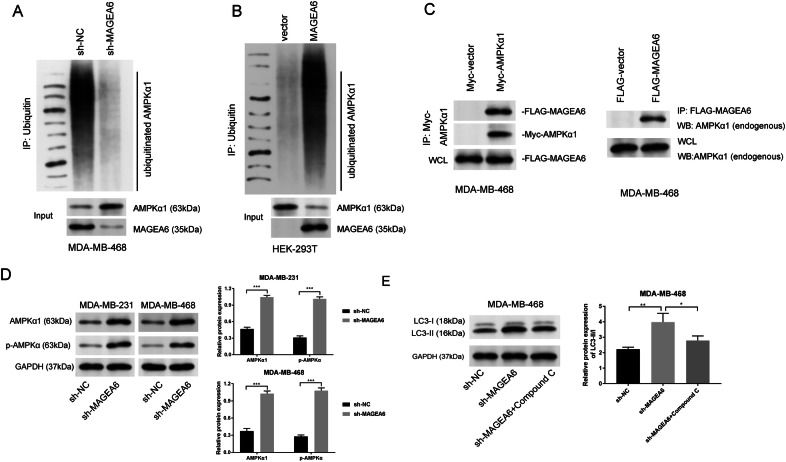
Inhibition of AMPK by MAGEA6 impacted the autophagy of TNBC. **A**, **B** In vitro ubiquitination assay displayed that AMPKα1 ubiquitination requires MAGEA6. **C** Co-immunoprecipitation assay stated the association between MAGEA6 and AMPKα1. **D** Western blotting showed that knockdown of MAGEA6 increased the levels of AMPKα1 and p-AMPKα (Thr-172). **E** Whether sh-MAGEA6 influenced autophagy via activating AMPK signaling was verified by Western blotting assay. *P < 0.05, **P < 0.01, ***P < 0.001; Data were obtained from three independent experiments. TNBC triple-negative breast cancer.

It’s well known that the MAGEA6-TRIM28/KAP1 complex is a specific ubiquitin ligase of AMPKα1 [18], and AMPKα1 is also closely relevant for mTOR to regulate autophagy [12]. In the current study, we further verified the TNBC. At first, in vitro, ubiquitination assay displayed that silencing MAGEA6 decreased the AMPKα1 ubiquitination in TNBC cells (Fig. 4A). Overexpression of MAGEA6 in HEK-293T cells accelerated the ubiquitination of AMPKα1 (Fig. 4B). Furthermore, overexpressed and endogenous AMPKα1 co-immunoprecipitated with MAGEA6 from cells (Fig. 4C). Correspondingly, knockdown of MAGEA6 caused the increases of AMPKα1 (Fig. 4D), suggesting that MAGEA6 modulated the AMPKα1 expression via ubiquitination pathway. Western blotting assay also detected a strong increase of p-AMPKα protein level in MAGEA6-silencing cells, suggesting that sh-MAGEA6 activated AMPK signaling, as determined by Thr-172 phosphorylation of AMPKα (Fig. 4D). To verify whether sh-MAGEA6 influenced autophagy via activating AMPK signaling, AMPK inhibitor (Compound C) was further added. Results displayed that Compound C can reduce the LC3-II/I level induced by sh-MAGEA6 (Fig. 4E). Thus, we believed that MAGEA6 silencing activated AMPK signaling through suppressing AMPKα1 ubiquitination, and then facilitated autophagy in TNBC.

### AMPK is required for SLC7A11 to regulate ferroptosis

**Fig. 5:**
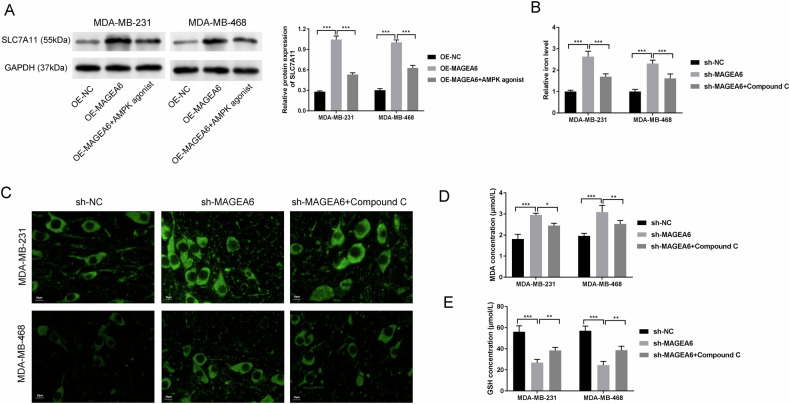
AMPK is required for SLC7A11 to regulate ferroptosis. **A** The influences of MAGEA6 combined with AMPK on SLC7A11 level in TNBC cells were examined by Western blotting assay. **B** The influences of MAGEA6 combined with AMPK on the iron level were shown. **C** The fluorescence intensity stained with C11-BODIPY 581/591 was used to evaluate the level of intracellular ROS; The contents of MDA (**D**) and GSH (**E**) in MDA-MB-231 and MDA-MB-468 cells were tested by ELISA assay; *P < 0.05, **P < 0.01, ***P < 0.001; Scale bar = 20 μm; Data were obtained from three independent experiments. TNBC triple-negative breast cancer.

SLC7A11 acts as a key repressor for ferroptosis and has previously been demonstrated to be mediated by the MAGEA6/AMPK axis [19]. To further investigate whether AMPK in TNBC cells is required for MAGEA6-regulated SLC7A11, we examined the influences of MAGEA6 combined with AMPK on SLC7A11 level in TNBC cells. As presented in Fig. 5A, overexpression of MAGEA6 increased SLC7A11 expression, whilst AMPK agonist (A-769662) reversed SLC7A11 expression. Next, we further examined the influences of MAGEA6 combined with AMPK on ferroptosis in TNBC cells exposure to erastin (a ferroptosis inducer). Results showed that total iron was notably elevated in MDA-MB-231-sh-MAGEA6 and MDA-MB-468-sh-MAGEA6 cells (Fig. 5B). Additionally, we used the FerroOrange probe to test the Fe^2+^ staining and noticed a distinct increase in fluorescence intensity of cells in the sh-MAGEA6 group (Fig. S2). In line, sh-MAGEA6 increased the level of intracellular ROS (Fig. 5C). Moreover, the content of GSH (a key substrate of GPX4), and MDA (a lipid peroxide product) were tested. The results disclosed that the MDA content in MDA-MB-231 and MDA-MB-468 cells increased with the down-regulation of MAGEA6, while the GSH content markedly decreased after MAGEA6 down-regulation (Fig. 5D, E). Importantly, AMPK inhibitors can rescue the above effect (Figs. 5B–E and S2). This evidence demonstrated that AMPK mediated by MAGEA6 is required for SLC7A11 to regulate ferroptosis in TNBC cells.

### MAGEA6/AMPK/SLC7A11-mediated ferroptosis influenced on TNBC resistance

**Fig. 6:**
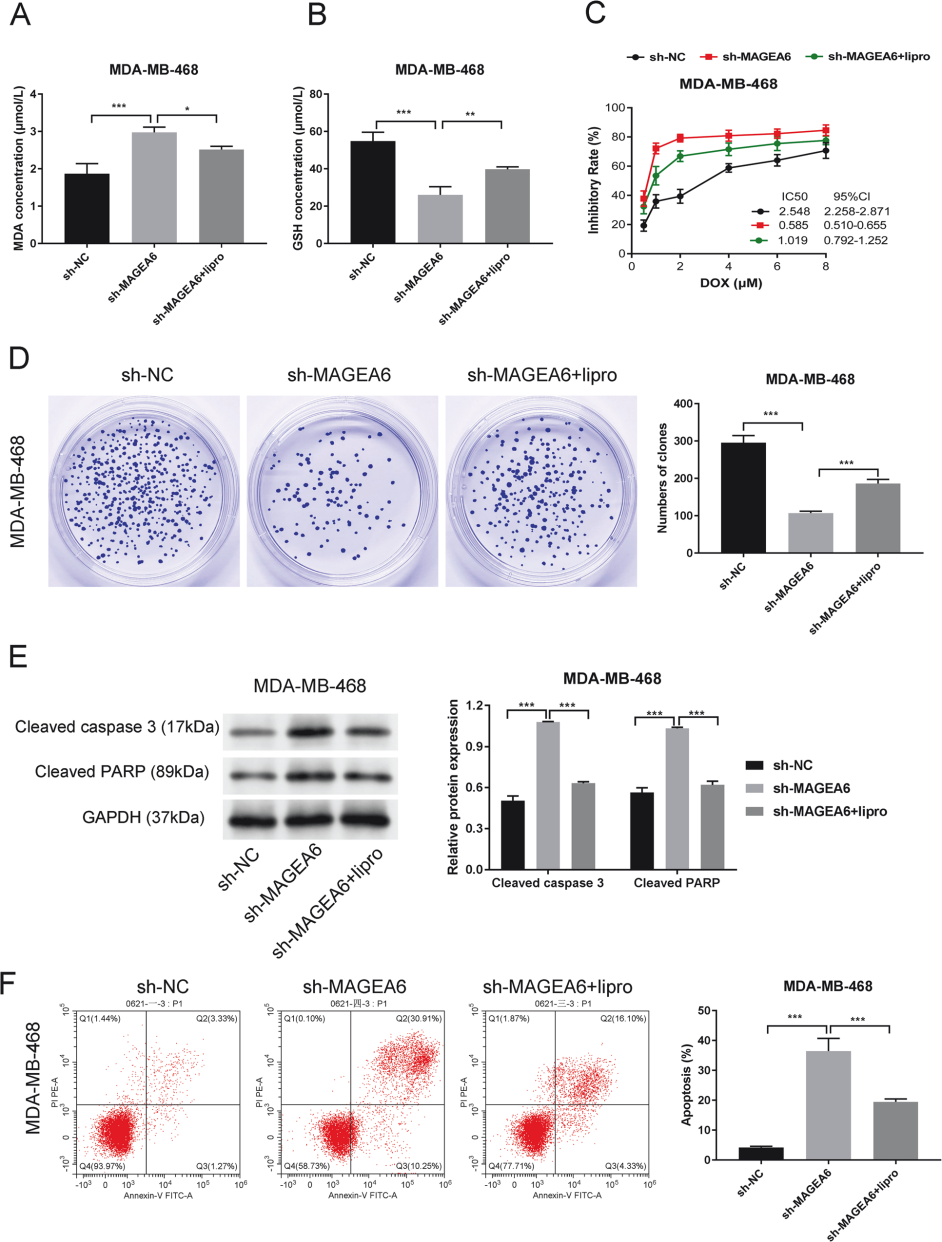
MAGEA6/AMPK/SLC7A11-mediated ferroptosis influenced on TNBC resistance. **A**, **B** ELISA results uncovered that ferroptosis inhibitors (liproxstatin-1, lipro) can rescue the MDA and GSH levels induced by sh-MAGEA6. **C** The influences of sh-MAGEA6 and lipro on IC_50_ value was determined by MTT assay. **D** Colony formation presented the proliferation ability among different groups. **E**, **F** The influences of sh-MAGEA6 and lipro on apoptosis in TNBC cells were examined by Western blotting and flow cytometry; *P < 0.05, **P < 0.01, ***P < 0.001. Data were obtained from three independent experiments. TNBC triple-negative breast cancer.

To determine the roles of ferroptosis modulated by MAGEA6/AMPK/SLC7A11 on the sensitivity of TNBC cells to DOX, a ferroptosis inhibitor (liproxstatin-1, lipro) was incubated (Fig. 6A, B). Then, MAGEA6 depletion-mediated IC_50_ value could be recovered by treatment with lipro (Fig. 6C). Also, the proliferation ability of sh-MAGEA6+lipro group was notably elevated by comparison with sh-MAGEA6 group (Fig. 6D); but corresponding apoptosis rate was reduced (Fig. 6E, F), supported the crux roles of MAGEA6/AMPK/SLC7A11-mediated ferroptosis on modulating DOX sensitivity in TNBC cells.

### MAGEA6 depletion increased the chemo-sensitivity of TNBC to DOX in vivo model

**Fig. 7:**
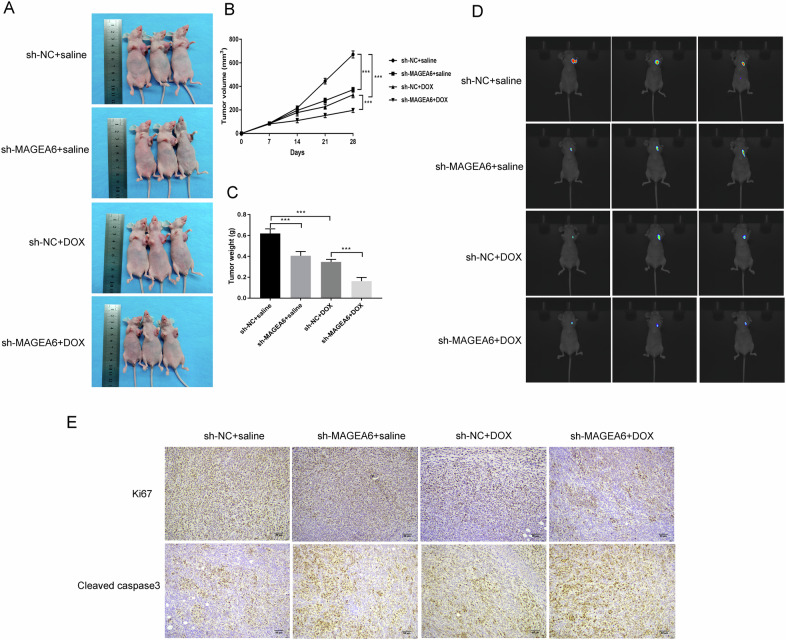
MAGEA6 depletion increased the chemo-sensitivity of TNBC to DOX in vivo model. **A**–**C** Tumor volume and weight were presented among different groups. **D** In vivo imaging assay uncovered the fluorescence intensity in the lung to evaluate the roles of sh-MAGEA6 combined with DOX on pulmonary metastasis. **E** Immunohistochemistry assay stated the influences of MAGEA6 depletion combined with DOX on the expressions of ki67 and Cleaved caspase3 in mice tumor tissues; ^***^P < 0.001; *n* = 3 mice; Scale bar = 80 μm. DOX doxorubicin. TNBC triple-negative breast cancer.

Further, we investigated the function of MAGEA6 on DOX sensitivity in vivo using a xenograft tumor model. As shown in Fig. 7A–C, tumor volume and weight were notably decreased in the sh-NC + DOX group as a comparison with the sh-NC + saline group (*P* < 0.001). Additionally, tumor growth was further remarkably reduced in the sh-MAGEA6 + DOX group (*P* < 0.001). Besides, in vivo imaging assay uncovered the fluorescence intensity in the sh-MAGEA6 + DOX group was lowest (Fig. 7D). In line with our results in vitro, MAGEA6 depletion combined with DOX leads to the remarkable decrease of ki67 expression, but increase of Cleaved caspase3 (Fig. 7E). Overall, our findings demonstrated that MAGEA6 depletion increased the chemo-sensitivity of TNBC cells to DOX in vivo model.

## Discussion

It is generally recognized that chemo-resistance is the main challenge for cancer treatment and contributes to cancer treatment failure [20]. So, enhancing the sensitivity of TNBC cells to chemotherapy would have survival benefits for TNBC patients. In our study, we identified a mechanism of MAGEA6 on chemo-resistance via influencing AMPKα1-mediated autophagy and ferroptosis in TNBC.

MAGEA6, as one of the MAGE family members, is a proto-oncogene whose elevated expression is involved in tumor growth and progression via affecting signaling pathways or potential mechanisms [21]. In line, prior studies have displayed the involvement of MAGEA in breast cancer progression, with potential implications for immunotherapy [22]. Besides, MAGEA6 serves as hub genes in the chemo-resistance of breast cancer [17]. In the current study, MAGEA6 was highly expressed in TNBC tissues and resistant-TNBC tissues, and relevant with poor prognosis. Silencing of MAGEA6 enhanced the DOX sensitivity of TNBC, as evidenced by decreases in IC_50_ value, reduction of invasion ability, and induction of apoptosis in vivo and in vitro. Importantly, MAGE has been adopted in clinical trials of metastatic melanoma [23]. This evidence suggested that inhibition of MAGEA6 may be a potential therapy to advance the efficacy of DOX for TNBC patients.

A recent study uncovers a role for MAGEA6 in the suppression of autophagy implicating MAGEA6 in tumorigenesis [24, 25]. Autophagy, as a highly conserved self-degradative process, becomes an element during tumor growth and therapy resistance in many cancers, including TNBC [26, 27]. Our study showed that MAGEA6 silencing remarkably decreased the amount of LC3-II/LC3I and autophagosomes. Furthermore, we also found that autophagy inhibitors (chloroquine, CQ) reversed the decreases of IC_50_ value caused by MAGEA6 depletion, indicating that MAGEA6 regulates tumor chemo-sensitivity via modulating autophagy.

Identifying the mechanisms of MAGEA6-mediated autophagy, Pineda et al. [12] have stated that MAGEA mediates the degradation of AMPK via ubiquitination, followed by down-regulating autophagy in cancer. Another report has demonstrated that MAGEA6 targets AMPKα1 and then affects cell survival [28]. Consistently, we described the molecular mechanism by which MAGEA6 functioned as repressors of autophagy via AMPKα1 ubiquitination. AMPKα1 is a catalytic subunit of AMPK, and its activation can drive autophagy to restrain cancer cells [29]. At the molecular level, our results showed a strong increase of p-AMPKα protein level in MAGEA6-silencing cells, suggesting that sh-MAGEA6-activated AMPK signaling. Considering the important function of AMPK in tumor-suppressing or chemo-resistance reducing [30], it is not surprising that AMPK signaling is often imbalanced in human cancers [31]. More importantly, AMPK inhibitor (Compound C) can reduce the LC3II/I level induced by sh-MAGEA6, which was detected in our study. Overall, we believed that MAGEA6 silencing activated AMPK signaling by suppressing AMPKα1 ubiquitination, and then facilitated autophagy to influence the chemo-sensitivity of TNBC cells.

Recently, FDA-approved drugs such as ferroptosis inducers are considered to be a new promising approach for cancer therapy [32]. Except for autophagy, prior studies have also reported the importance of AMPK’s role in ferroptosis of cancer progression [33–35]. In the regulation of the ferroptosis process, our study stated that AMPK is required for SLC7A11 to regulate ferroptosis, consistent with a prior study that SLC7A11 acts as a key repressor for ferroptosis mediated by the MAGEA6/AMPK axis [19]. It’s well known that SLC7A11 functions as a core modulator of ferroptosis in multiple human cancers, including TNBC [36, 37]. Regarding SLC7A11-mediated ferroptosis, accumulating evidence has verified that SLC7A11 overexpression could confer resistance to chemotherapeutic drugs, radiotherapy, or immunotherapy through restraining ferroptosis [38, 39]. Accordingly, we further observed that ferroptosis inhibitors (liproxtatin-1, lipro) can rescue the MAGEA6 depletion-mediated proliferation ability, apoptosis, and IC_50_ value, supporting the crux roles of MAGEA6/AMPK/SLC7A11-mediated ferroptosis on modulating DOX sensitivity in TNBC cells.

Our study illustrated for the first time a potential mechanism that MAGEA6 mediated the degradation of AMPKα1 through ubiquitination influences the autophagy and ferroptosis, and then enhances chemo-resistance. Given the importance of autophagy and ferroptosis as master regulator in human cancer, MAGEA6/AMPKα1 may provide insights into slowing cancer cell progression and therapeutic targets.

## Material and methods

### Microarray data collection

GSE26459 and GSE16179 datasets comparing resistant breast cancer cells and sensitive breast cancer cells were downloaded from the Gene Expression Omnibus (GEO). To analyze the differential expression genes (DEGs) in normal and tumor tissues, Deseq2 package (1.42.0) was used on the basis of TCGA (The Cancer Genome Atlas) datasets. Genes with |log2 fold change (FC)| > 1 and *P* value < 0.05 were identified as DEGs. Kaplan–Meier plots of MAGEA6 (214612_at) on survival probability of breast cancer patients, including overall survival (OS) and relapse-free survival (RFS), were analyzed by kmplotter online tool (https://kmplot.com/analysis/).

### Clinical samples

Two independent cohorts of TNBC patients were included in this study. Cohort 1 consisted of 30 tumor samples and adjacent non-tumor tissues from TNBC patients who had not received chemotherapy or radiotherapy. Cohort 2 consisted of 36 TNBC patients who received DOX chemotherapy post-surgery. Among them, 12 patients manifesting relapse within 6 months after the last course of chemotherapy were defined as the DOX-resistant group, and 24 patients with no recurrence during follow-up were defined as the DOX-sensitive group, according to a previous study [40]. The study was approved by the Ethics Committee of China–Japan Union Hospital of Jilin University (2024-KYYS-098). Informed consent was obtained from all subjects.

### Immunohistochemistry assay

Tumor tissues were fixed with 4% paraformaldehyde, dehydrated, embedded into paraffin, and incised into 4 μm sections. The slides were exposed to 3% hydrogen peroxide-containing methanol, followed by incubating with 10% goat serum. After washing, the sections were incubated with primary antibodies against MAGEA6 (PA5-51255, 1:50), Ki67 (PA5-19462, 1:200) (Invitrogen, Carlsbad, CA, USA), and Cleaved caspase3 (25128-1-AP, 1:150, Proteintech, Wuhan, China) and then with peroxidase-labeled IgG. Next, the sections were dyed with 3,3’-diaminobenzidine (DAB) and visualized by microscopy (CX31, Olympus, Tokyo, Japan).

### Cell culture

Triple-negative breast cancer cell lines (MDA-MB-231 and MDA-MB-468) and normal breast epithelial cells (MCF 10A) were purchased from Procell (Wuhan, China). Cells were cultured in DMEM (Procell, Wuhan, China), supplemented with 10% FBS (Procell, Wuhan, China) and 100 U/ml streptomycin/penicillin (Transgen, Beijing, China). Moreover, a Mycoplasma Detection Kit-Quick Test (iNtRON, Shanghai, China) was employed to verify that cells were not contaminated with mycoplasma.

### Cell transfection

Lentiviral constructs of human MAGEA6 silencing (sh-MAGEA6) and its corresponding vector (sh-NC) were designed by Sangon Biotech Co., Ltd (Shanghai, China). Subsequently, Lipo6000™ Transfection Reagent (Beyotime, Shanghai, China) was utilized to transfect the sh-MAGEA6 into MDA-MB-231 and MDA-MB-468 cells. After transfection, cells were treated with DOX for 24 h and then collected for the next experiments.

### RT-qPCR assay

Total RNA was extracted with TRIzol Reagent, and then RT-qPCR was conducted using a one-step universal PCR kit (Tiangen, Beijing, China). Primer sequences were listed as follows: MAGEA6 (Forward): 5’-CCAGATCCTCCCCAGAGTC-3’, (Reverse): 5’-TGAACCAACTTGGCCACCTT-3’; GAPDH (Forward): 5’-CTGGGCTACACTGAGCACC-3’, (Reverse): 5’-AAGTGGTCGTTGAGGGCAATG-3’; GAPDH was regarded as an internal reference for quantifying mRNA expressions.

### Western blotting

Cells were lysed in RIPA lysis buffer (CWBIO, Beijing, China), and the protein concentration was tested by a BCA kit (CWBIO, Beijing, China). Afterwards, the protein (30 μg) was separated and electrically transferred to the PVDF membrane. The membranes were blocked with 5% skimmed milk, followed by incubating overnight with antibodies against MAGEA6 (PA5-75647, 1:500, Invitrogen, Carlsbad, CA, USA), LC3 (14600-1-AP, 1:3000), Cleaved caspase 3 (25128-1-AP, 1:1500), Cleaved PARP (13371-1-AP, 1:5000), AMPKα1 (66536-1-Ig, 1:3000), SLC7A11 (26864-1-AP, 1:1500) (Proteintech, Wuhan, China); and p-AMPKα (Thr-172) (2531, 1:1000, CST, Boston, MA, USA). After washing, anti-Rabbit IgG-HRP (1:5000, YEASEN, Shanghai, China) were incubated, and immunoreactions were visualized by the enhanced chemiluminescence (ECL) kit. With GAPDH as an internal reference, the protein levels were analyzed by Image J software.

### Flow cytometry assay

For determining the cell apoptosis, MDA-MB-231 and MDA-MB-468 cells (1 × 10^6^ cells) were obtained after transfection combined with DOX treatment. Cell apoptosis was detected using Annexin V-propidium iodide (PI) Kit (Amyjet Scientific, Wuhan, China) on flow cytometer.

### MTT assay

For the IC_50_ assay, MDA-MB-231 and MDA-MB-468 cells (1 × 10^4^ cells) were treated with DOX or DMSO after cell incubation and attachment, and then MTT reagent (5 mg/ml) was added. Following incubation, the absorbance was determined by a microplate reader (Infinite EPlex, Tecan, Switzerland).

### Colony formation assay

MDA-MB-231 and MDA-MB-468 cells (1 × 10^3^ cells) with designated treatment were inoculated into a culture disk. After incubation for 14 days, cells were fixed by methanol and stained with 0.2% crystal violet for 15 min.

### Transwell assay

MDA-MB-231 and MDA-MB-468 cells (1 × 10^5^ cells) were inoculated in the upper chamber with Matrigel-coated inserts. After incubation, cells were fixed with methanol and stained with 0.2% crystal violet. Images were captured by microscope (CX31, Olympus, Tokyo, Japan), and the numbers of cells were visualized by Image J software.

### mRFP-GFP LC3 autophagy assay

After mCherry-GFP-LC3 transfection, cells were washed and then incubated with Earle’s balanced salt solution (EBSS) for the indicated durations. Following, cells were fixed and delivered to confocal microscopy for analysis.

### In vitro ubiquitination assay

MDA-MB-231 and HEK-293T cells were transfected with silencing or overexpressing MAGEA6 plasmid constructs along with HA-tagged ubiquitin, and then administrated with 10 μM MG-132 for 6 h. Whole cell lystates (WCL) were utilized for immunoprecipitation (IP) with anti-AMPKα1 overnight. The AMPKα1 ubiquitination was evaluated by immunoblotting with anti-ubiquitin (ARG54630, Arigo Biolaboratories, Taiwan, China) antibody.

### Co-immunoprecipitation assay

MDA-MB-231 and HEK-293T cells were co-transfected with FLAG-tagged MAGEA6 and Myc-tagged AMPKα1 (with empty vector) individually or in combination. Cell lysates were obtained and immunoprecipitated with anti-FLAG or anti-Myc antibodies.

### ELISA assay

The concentrations of MDA and GSH were quantified using an ELISA kit (Solarbio, Beijing, China). The absorbance was measured by a microplate reader (Infinite EPlex, Tecan, Switzerland).

### Iron assay

The total iron of TNBC cells was analyzed using an iron assay kit (Sigma-Aldrich, St. Louis, MO, USA) in accordance with the manufacturer’s procedure.

### Fe^2+^ staining analysis

To test the intracellular Fe^2+^ level in MDA-MB-231 and MDA-MB-468 cells, FerroOrange working solution (1 mol/l, BIOFOUNT, Beijing, China) was added, and the cells were incubated for 30 min in darkness, as per the manufacturer’s instruction. Next, cells were observed under a fluorescence microscope (Lecia, Germany).

### Lipid reactive oxygen species (ROS) detection

To determine Lipid ROS, MDA-MB-231 and MDA-MB-468 were incubated C11-BODIPY 581/591 (2 μM, Shanghai Maokang Biotechnology Co., Ltd, Shanghai, China) for 1 h. Then, the washed cells were subjected to detect the fluorescence intensity of a microscope (Lecia, Germany).

### Xenograft tumor model in vivo

BALB/c nude mice (6 weeks, Charles River, Beijing, China, *n* = 24) were randomly divided into four groups. For the in vivo tumor growth assay, MDA-MB-468 cells (1 × 10^6^ cells) were injected into breast tissue from the lower left leg of BALB/c nude mice (6 weeks, Charles River, Beijing, China, *n* = 3). One week after injection, the mice were given DOX (4 mg/kg) once a week, and the control group was injected with normal saline for 4 weeks. Tumor formation was monitored, and the mice were sacrificed 4 weeks post-inoculation.

For pulmonary metastasis assay, MDA-MB-468 cells (1 × 10^6^ cells) were injected into the tail vein of BALB/c nude mice (*n* = 3). Animal experiments were conducted according to animal care and use guidelines and approved by the Animals Ethics Committee of the Third Military Medical University.

### Statistical analysis

Data were presented as mean ± standard deviation (SD) and analyzed using the GraphPad Prism 7. The variances between the two groups were compared with the Student’s *t*-test, while variances among multiple groups were analyzed by one-way ANOVA. *P* < 0.05 was termed as statistical significance.

## Supplementary information

Figure S1

Figure S2

supplementary file-WB

## Data Availability

The data that support the findings of this study are available from the corresponding author upon reasonable request.
