# Supplementary figures and images for: Targeting oncogenic MAGEA6 sensitizes triple negative breast cancer to doxorubicin through its autophagy and ferroptosis by stabling AMPKα1
Source: Cell Death Discov. 2024 Oct 6;10:430. doi: 10.1038/s41420-024-02196-9 (PMC11456603; doi:10.1038/s41420-024-02196-9)

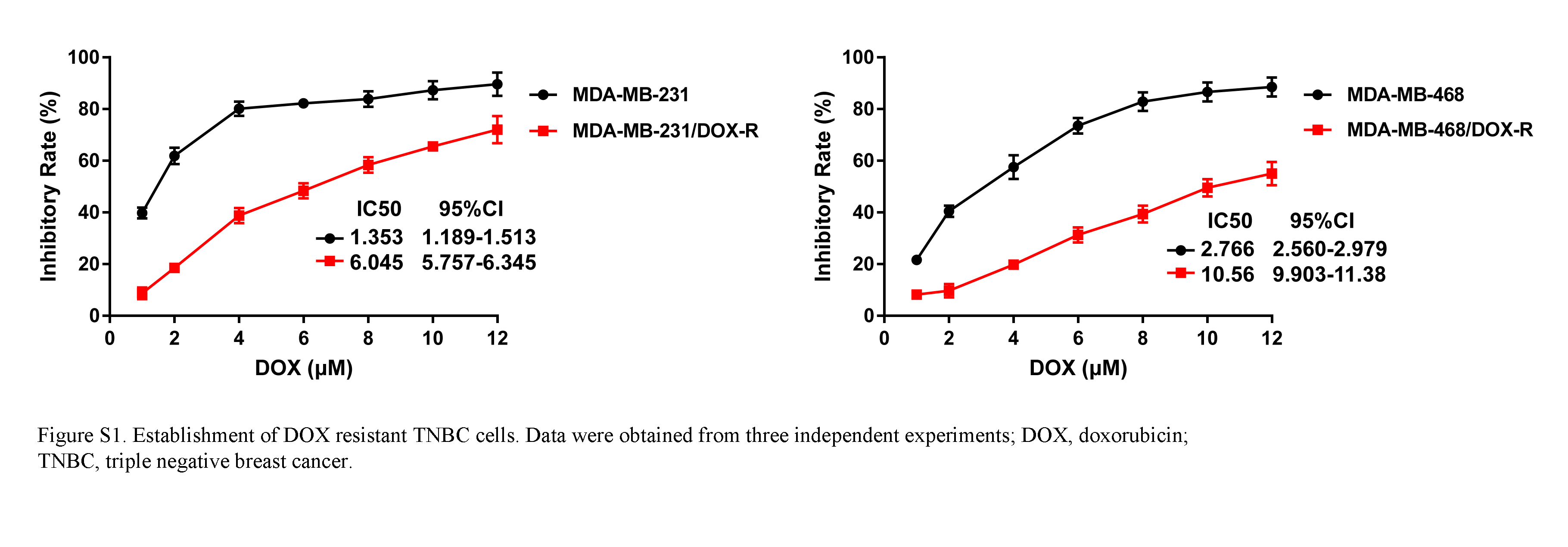

Supplement: Supplementary file 1 — Figure S1 [file 41420_2024_2196_MOESM1_ESM.tif]
