# Supplementary material for: Targeting oncogenic MAGEA6 sensitizes triple negative breast cancer to doxorubicin through its autophagy and ferroptosis by stabling AMPKα1
Source: Cell Death Discov. 2024 Oct 6;10:430. doi: 10.1038/s41420-024-02196-9 (PMC11456603; doi:10.1038/s41420-024-02196-9)
Supplement: Supplementary file 3 — supplementary file-WB [file 41420_2024_2196_MOESM3_ESM.docx]

fig 1F







fig 1G







fig 2E















fig 3A











fig 4A









fig 4B









fig 4C-1









fig 4c-2







fig 4D















fig 4E







fig 5A











fig 6E
